# Thermal Osteonecrosis Caused by Bone Drilling in Orthopedic Surgery: A Literature Review

**DOI:** 10.7759/cureus.5226

**Published:** 2019-07-24

**Authors:** Charles Timon, Conor Keady

**Affiliations:** 1 Orthopaedic Surgery, National University of Ireland, Galway, IRL; 2 Orthopaedics, University Hospital Galway, Galway, IRL

**Keywords:** thermal osteonecrosis, bone drilling, orthopaedic surgery

## Abstract

Thermal osteonecrosis is the in situ death of bone tissue as a result of excessively high temperatures. While the exact temperature at which thermal osteonecrosis occurs has not yet been determined, 50°C is the accepted critical value, as bone regeneration is almost completely impaired from this point on. Thermal osteonecrosis is a significant concern in orthopedic surgery, as it can compromise the bone-implant interface in fracture fixation, which, by definition, is a complication.

A literature review was undertaken of the pertinent literature concerning heat generation from bone drilling and how this heat affects bone tissue. The Pubmed, ScienceDirect, and secondary (Cochrane Library) databases were searched up to December 2017 using keywords with the appropriate use of Boolean operators. Both simple text word searching and thesaurus searching were used to maximize the number of relevant articles retrieved. Reference tracking was performed via the retrieved articles to further extend the boundaries of the search. The level of evidence was Level V.

It was identified that factors affecting heat generation during bone drilling were multifactorial and did not act independently of each other. Good quality evidence exists that both bone drilling parameters and the drill itself affect heat generation in bone during bone drilling. However, external irrigation is the most important variable and should always be used to keep the bone temperature below the critical value of 50°C.

Future studies should focus on how the parameters of bone drilling interact with each other and how this influences heat generation in bone drilling. There is also a lack of in vivo studies on the human bone; this too should be further investigated.

## Introduction and background

The treatment of bone fractures has existed ever since the practice of medicine began. In the practice of modern medicine, fractures are managed with either the conservative or surgical approach. Surgical management involves fixing the reduced fracture by drilling screws and plates to immobilize the fracture. Thermal osteonecrosis is a well-described phenomenon and is a significant concern in trauma and orthopedic surgery [1]. Thermal osteonecrosis is the phenomenon of in situ bone death as a result of excessively high tissue temperatures. In orthopedic surgery, high-speed bone drilling is the predominant cause of thermal bone necrosis. Most of our knowledge regarding bone drilling is adapted from the fields of dentistry and orthodontics. Eriksson and Albrektsson found that bone heated at 47°C for one minute leads to significantly reduced bone regeneration and that bone temperatures of 50°C almost completely impairs it [2]. Cell death as a result of heat damage is immediately apparent at 70°C [3-4]. Krause’s findings support this, he found that osteoclasts began to die when bone temperatures reached 50°C and that protein cannot be regenerated once temperatures reach 70°C [5]. As a result of this, 50°C has been defined as the critical value below which bone temperature must be kept.

Bone drilling is a significant part of the daily practice of modern-day orthopedic and trauma surgery. Compression fixation in fracture management requires enduring stability of the fixating screws. Heat arising from these operations may cause thermal necrosis of bone. With regards to current fracture fixation principles, every loosening of a bone implant must be defined as a complication. Deficient drilling may even cause weakening and breaking of the implant from fixation sites (screws and pins) [6]. Lower leg reduction and internal fixation have a failure rate of 2%-7% according to the literature [7].

Many factors can lead to the compromise of bone-implant interfaces, thermal osteonecrosis being just one of them. The bone temperature must be kept below the temperature of 50°C to ensure thermal osteonecrosis does not occur [8-9]. Many factors have been studied in an attempt to reduce heat generation; this includes alternative drill designs, drilling parameters, and cooling techniques. Despite the large number of variables that contribute to temperature generation, most of the published literature only studies one or several of the involved factors in this complicated issue. This has led to discrepancies in the ideal combination of drill design, drilling speed and technique, and cooling techniques.

Additionally, the optimal technique for measuring bone temperature is difficult to determine. This is because bone is a complex biological tissue composed of organic and inorganic materials. These materials interact, resulting in complex thermal and physical properties, making the tissue challenging to study due to its sensitivity to specimen preparation and conditions [10].

## Review

Bone drilling parameters

These factors do not influence bone temperature changes independently but, in fact, are all interrelated. This is a large flaw in much of the literature investigating thermal osteonecrosis and bone temperature changes and must be considered when discussing any results yielded [11]. Additionally, the absolute values of temperature rise need to be considered carefully, accounting for the study design, i.e. was the experiment executed at room temperature or at body temperature in a controlled solution? And whether a substitute for the cooling effect of local blood flow was used or not? However, for the purpose of this paper, we will discuss both the parameters of the drill and the parameters of bone drilling itself.

Parameters of the Drill

Drill design: Flutes are the groves in the body of a drill that provide cutting lips. This is to facilitate the removal of chips and to allow cutting fluid to reach the lips. Bertollo et al. showed that three-fluted drills had significantly higher feed rates than two-fluted drills. As feed-rate is proportional to cutting efficiency, this suggested that the three-fluted drills had a higher cutting efficiency reduction of heat generation than the two-fluted. Despite this, the three-fluted drills did not, in fact, result in a reduction in heat generation or in decreased rates of screw loosening [12].

Drill diameter: This has been shown to correlate with bone temperature exponentially [7]. This is despite the fact that larger drills have larger flutes, which facilitate more efficient drilling as they are better at eliminating bone chips. However, smaller drills are weaker and can bend within the bone, leading to surgeons missing their targets [13].

Drill wear and tear: This is another factor that must be taken into account [14]. Allan examined three of the same drills that had been in use for different periods of time and found that temperatures generated varied significantly.

Parameters of Bone Drilling

Drill speed: There is no generally accepted consensus on optimal drilling speed. Early studies found that bone temperature was proportional to drilling speed [15]. However, this is only true up to speeds of 10,000 rpm as confirmed by later studies [16]. One study found no changes in temperature from drilling speeds of 345 rpm up to 2900 rpm from drilling human cadaveric bone. This implied that temperature increase was more dependent on drilling pressure rather than on speed. Sezek concluded that a rotation speed of 370 rpm and a feed rate of 70 mm/min were optimal values for a safe and successful drilling process [17]. Brisman showed that drilling bovine cortical bone at a low speed and minimal pressure produced the same temperature increase as drilling at high speed and with a higher pressure load. This is because increasing speed and pressure together allows for more efficient cutting of bone [18]. Furthermore, Thompson’s original study showing that drilling speed is proportional to temperature increase also found that speeds of less than 250 rpm caused increased fragmentation around the surface of the drilling holes, further proving the importance of bone drilling parameters in ensuring successful osteosynthesis.

Feed rate: The drill feed rate is an important parameter in determining the heat generated during bone drilling. As the feed rate increases, drilling time decreases and less heat is generated. However, as a higher force is applied during drilling, pressure increases, which can cause increased heat generation. Because of this, it is important to determine the optimal feed rate. The general consensus derived from the literature is that increasing axial force significantly reduces drilling time, bone temperature time above 50°C, and maximum bone temperature [19]. Nam et al. found that either the combination of low speed (600 rpm) and high force (1000 g) or high speed (1200 rpm) and low force (500g) to be optimal, resulting in temperatures of 40°C-45°C, thus staying below the critical value of 50°C.

Drilling depth: Bone temperature increases with drilling depth [20]. Kalidindi showed that deeper drilling results in longer contact time between the moving drill bit and bone; this increases the friction generated, resulting in an increase in the amount of heat generated [21]. Deeper drilling causes a significant increase in bone temperature. regardless of the drill-bit diameter and cooling systems used [22].

Predrilling: Predrilling is the practice of incrementally increasing the drill diameter in the same hole. The theory behind this is to gradually increase the size of the hole and allow better dissipation of the heat generated by the drilling. The main drawback of this technique is increased operative time. In addition, maintaining the same direction as the pre-drilled hole may prove problematic. Kalidindi proved that the maximum temperature reached using pre-drilling is significantly less than that reached when drilling the final diameter hole in a single attempt [21].

Bone thickness: The cortex is the hardest part of the bone; the time taken during bone drilling depends on the thickness of the cortex. The hardness of the cortical bone is proportional to bone mineral density (BMD). It has been shown that the higher the BMD, the more heat generated by bone drilling when all other parameters of drilling are equal [23]. In order to achieve stable reduction and internal fixation, both pieces of cortical bones must be drilled to attach the chosen fixator. The human femur has a cortical bone thickness of approximately 6-6.5 mm and the average drilling time is 18 seconds [9]. Sezek et al. concluded that pre-operative bone mineral density should be obtained and that this should be used to select the applied drill force, feed rate, and drill speed. By doing this, one could optimize the drill parameters, and this could help minimize the necrosis, which complicates bone fixation operations in orthopedic surgery [17].

Irrigation: Augustin et al. showed us that the most important variable in avoiding thermal osteonecrosis during bone drilling is the use of external irrigation [7]. Throughout all variations in drill diameter and speed etc., external irrigation kept the bone tissue temperature below the critical value of 47°C (range 29.9°C - 33.9°C). Without external irrigation, all other parameters being the same, bone temperature ranged from 31.4 C to 55.5°C. While they acknowledged and explored the myriad of variables that influence bone temperature during drilling, they concluded that external irrigation with saline is the most important factor in preventing increases in bone temperature during drilling and went so far as to declare external irrigation mandatory.

Temperature measurement

Method

Two methods exist with which one can measure bone temperature during drilling: infrared cameras and thermocouple devices. There is a small amount of data where both methods are used and there exist no studies comparing both methods.

A thermocouple is a device containing two different conductor probes. It works by producing a voltage proportional to the temperature difference between either end of the pair of probes. Two or more thermocouples can be used simultaneously to analyze temperature dissipation through the bone [24]. Usually, thermocouples are placed 0.5 mm and 1 mm from the drilling site, which is suitable for analyzing results.

Predicting temperature dissipation in bone requires the use of complex equations and many parameters such as the geometry, specific heat, and thermal conductivity of bone. To simplify this process, as well as to avoid the likely errors resulting from such complex equations, the thermographic infrared gun was invented. It was first developed by DaimlerChrysler, the American automobile manufacturer in the early ‘90s. It was adapted for orthodontic use in 2006 [16]. Infrared thermography can be used to observe the 2D distribution of temperature throughout the bone. Combining thermographic infrared cameras perpendicularly cannot be used to assess 3D bone temperature, as it will still only measure surface values. The main disadvantages of thermography are that it is indirect so certain values must be extrapolated. Furthermore, thermographic cameras should be calibrated so a thermocouple may still be required [25].

Experiment setup

For the purpose of reviewing the literature, the experimental setup is an issue. Rarely are the experiments conducted intra-operatively on humans; most are conducted on animal models or cadavers. Porcine and canine bone has been shown to be most similar to human samples [9], however, Aerssens et al. found that there are considerable differences in the physiological properties of different species’ bone [26]. Furthermore, one must take into account the large variability in human bone properties among people of different ages, genders, race, etc. Kalidindi compared poly-methyl methacrylate (PMMA), commonly known as bone cement, to human bone because of their thermal properties. He showed that they have similar thermal conductivity but that human bone achieves maximum temperatures of 15%-30% higher than PMMA when subjected to the same conditions [21]. This is because human bone has higher thermal diffusivity due to the fact that it is denser than PMMA. Despite this, Kalidindi determined that PMMA is an adequate alternative to human bone for the purpose of bone drilling research. Some of the experiments on bone drilling were carried out in containers filed with saline solutions maintained at physiological temperature. The idea was that the solution would aid in heat transfer and prevent dead space from interfering with results. Bone wax was used as a sealant to prevent any of the fluid from entering the hole and tampering with results. The disadvantages of this method are the possibility of saline leakage and the loss of bone-air heat transfer, as would happen in an orthopedic setting.

Thermal osteonecrosis

Osteonecrosis is defined as the death of in situ bone tissue due to loss of blood supply [27]. It can be seen on X-ray as ring sequestrate encircling the implant. It was first described by Brock, in 1925, when he wrote about the decreased viability of bone caused by local frictional heat generated in orthopedic procedures. He wrote that infection and corrosion were the main causes of this reduced viability and that thermal heat damage was only a minor factor. The term ‘aseptic necrosis’ was coined in 1943 by Anderson and Finlayson after they noticed that orthopedic pin insertion burnt local bone [28]. Live measurement of heat generation was first carried out in 1992 by Watanabe et al. when they used infrared thermography to detect possible thermal osteonecrosis on porcine ribs [29]. This was first done in vivo on human patients by Olson et al. in 2011 [30].

The exact point at which thermal osteonecrosis occurs to human bone is yet to be determined. Temperatures above 70°C have been shown to cause immediate bone cell death (31). Irreversible enzymatic changes to cortical bone were observed at 50°C. Erikkson and Albrektsson found that heating titanium implants in rabbit bone to 50°C for one minute caused 30% of the bone to be resorbed and eventually replaced by fat cells; this prevented bone cells from growing into the porous implant, compromising its stability [2-3,8]. They also found that heating bone to 47°C for one minute reduced the amount of bone that grew into the implant.

Oschner et al. graded thermal osteonecrosis into four levels following intramedullary reaming [31]. Grade 0 results in no damage, devascularisation, or heat-induced damage. In Grade 1, the heat-damaged zone is cut away during the reaming; the only remaining damage is devascularization. In Grade 2, the damaged bone is not fully removed by reaming and there are devascularisation and head-damaged bone. In Grade 3, the entire cross-section of the bone, including the periosteum is devitalized by exposure to excessively high temperatures. He also concluded that the presence of thermal osteonecrosis is of vital importance in the presence of infection and may lead to grave consequences [32]. Follow-up studies recommended the practice of ‘ream-to-fit’ and of increasing the amount of time between successive reamers to allow the heat generated to dissipate, and, in theory, reduce the risk of causing thermal osteonecrosis [33].

It is important to note that blood flow is present in in-vivo experiments, be they on human or animal models, Field and Sumner-Smith research blood flow to the bone following bone drilling and noticed significant ischemia in the area directly surrounding the drilled holes [34]. Local perfusion is, in addition, compromised by the fracture itself, which, if it disrupts local arterial blood flow, can contribute further to the degree of thermal osteonecrosis. Cortical blood flow in vivo may allow the dissipation of some of the heat produced by drilling but, in reality, this is likely not to have any significant effect. Matthews and Hirsch compared temperature levels when drilling human femurs in vivo and in vitro and confirmed that blood flow does not significantly affect local temperature during bone drilling [35].

## Conclusions

This review aimed to analyze the most significant factors concerning heat generation during orthopedic bone drilling. There is still a paucity of data regarding some factors and conflicting findings, both of which need to be more closely assessed. The lack of research involving in vivo human patients was particularly surprising and may yield important findings in the future.
